# Survey of severe sepsis and septic shock management in Thailand: THAI-SHOCK SURVEY 2013

**DOI:** 10.1186/cc13270

**Published:** 2014-03-17

**Authors:** K Chittawatanarat, B Patjanasoontorn, S Rungruanghiranya, Thai SCCM Study Group

**Affiliations:** 1 Faculty of Medicine, Chiang Mai University, Chiang Mai, Thailand; 2 Khon Khaen University, Khon Khaen, Thailand; 3 Srinakharinwirot University, Nakornnayok, Thailand; 4 Thai Society of Critical Care Medicine, Bangkok, Thailand

## Introduction

A pragmatic survey of shock management in Thai physicians is unavailable. The objective of this study is to identify shock management patterns for severe sepsis and septic shock in Thailand.

## Methods

Two thousand questionnaires were sent to physicians involved in caring for shock patients across Thailand. The frequency scale was defined as five levels by patient proportion estimation at routine practice.

## Results

Between April and August 2013, a total of 533 questionnaires (26.7%) were returned. For severe sepsis and septic shock management, a total of 406 physicians (76.2%) reported their routine usage of quantitative resuscitation protocols. Urine output, mean arterial pressure and central venous pressure are more frequently used than central venous oxygen saturation and lactate as the resuscitation endpoint. Nearly 80% of these had an 'often and always' resuscitation goal within 6 hours. Most physicians (65.3%) never used procalcitonin. The antimicrobial empirical treatments were started within 1 hour for 87.7% and these were continued less than 5 days in 67.3% before deescalation. Crystalloid was the common initial fluid therapy in 98.9%. The most common used vasopressor was norepinephrine (69.6%). The median of cortisol threshold level for steroid replacement therapy was 15 (interquartile range, 5 to 19) mg/dl. Almost all of the physicians used hydrocortisone (96.4%). The median daily dose of hydrocortisone was 300 mg (interquartile range, 200 to 300). Nearly 50% divided the dose every 8 hours and 31.8% infused continuously. The duration for tapering was varied (33.6% in 2 to 3 days). Central venous pressure (CVP) and fluid challenge test were more frequently used for preload evaluation than new fluid responsiveness methods. Less than 15% still used a pulmonary artery catheter in their routine practice.

## Conclusion

Most physicians manage shock with protocols. Hemodynamic endpoints are preferred to tissue perfusion targets. Early antimicrobial therapy and de-escalation are routine practices without use of infective biomarkers. Crystalloid is preferred rather than colloid at initial resuscitation. CVP and fluid challenge are still more popular than new fluid responsiveness methods on preload assessment. Hydrocortisone is the most common steroid prescription in septic shock but the threshold of initiation, frequency and discontinuation are varied.

